# Clinical Studies of Nonpharmacological Methods to Minimize Salivary Gland Damage after Radioiodine Therapy of Differentiated Thyroid Carcinoma: Systematic Review

**DOI:** 10.1155/2016/6795076

**Published:** 2016-06-30

**Authors:** Andri Christou, Evridiki Papastavrou, Anastasios Merkouris, Savvas Frangos, Panayiota Tamana, Andreas Charalambous

**Affiliations:** ^1^Department of Nursing, School of Health Sciences, Cyprus University of Technology, 3036 Limassol, Cyprus; ^2^Thyroid Cancer Unit, Nuclear Medicine Department, Bank of Cyprus Oncology Center, 2006 Nicosia, Cyprus; ^3^University of Turku, 20014 Turku, Finland

## Abstract

*Purpose.* To systematically review clinical studies examining the effectiveness of nonpharmacological methods to prevent/minimize salivary gland damage due to radioiodine treatment of differentiated thyroid carcinoma (DTC).* Methods.* Reports on relevant trials were identified by searching the PubMed, CINHAL, Cochrane, and Scopus electronic databases covering the period 01/2000–10/2015. Inclusion/exclusion criteria were prespecified. Search yielded eight studies that were reviewed by four of the present authors.* Results.* Nonpharmacological methods used in trials may reduce salivary gland damage induced by radioiodine. Sialogogues such as lemon candy, vitamin E, lemon juice, and lemon slice reduced such damage significantly (*p* < 0.0001, *p* < 0.05, *p* < 0.10, and *p* < 0.05, resp.). Parotid gland massage also reduced the salivary damage significantly (*p* < 0.001). Additionally, vitamin C had some limited effect (*p* = 0.37), whereas no effect was present in the case of chewing gum (*p* = 0.99).* Conclusion.* The review showed that, among nonpharmacological interventions, sialogogues and parotid gland massage had the greatest impact on reducing salivary damage induced by radioiodine therapy of DTC. However, the studies retrieved were limited in number, sample size, strength of evidence, and generalizability. More randomized controlled trials of these methods with multicenter scope and larger sample sizes will provide more systematic and reliable results allowing more definitive conclusions.

## 1. Introduction

According to the American Thyroid Association (ATA) [1], radioiodine treatment of differentiated thyroid cancer (DTC) has three goals: (1) for remnant ablation, to facilitate detecting recurrent disease, (2) as adjuvant therapy to destroy remaining thyroid cancer cells, minimizing recurrence risk, and (3) as a means to address persistent disease reflected by thyroglobulin (Tg) levels. When such levels are high, radioiodine therapy (RAIT) is strongly recommended.

Despite being reasonably safe [1], radioiodine therapy (RAIT) is not always without side effects. Sialadenitis is the most common side effect with RAIT [2, 3], with an incidence rate ranging from 24% to 67% treatment [4]. Sialadenitis is a disease of the parotid, submandibular, and sublingual salivary glands, particularly of the first two of these sites [5]. The condition causes pain and swelling leading to oral discomfort [2, 6].

^131^I may damage the salivary glands by increasing their vascular permeability. The increased permeability allows plasma proteins to enter the saliva along with electrolytes above and beyond the sodium and chloride produced by the acinar cells normally transported in that fluid. As a result, biochemical changes, namely, elevated sodium and chloride concentrations and reduced phosphate levels, can be noticed in the saliva [7]. The toxic effect of ^131^I on the salivary glands may be quite serious, due to these glands' ability to absorb high levels of iodine compared to other tissues [6].

Another side effect of therapy that is related to salivary gland damage is dry mouth (xerostomia), which results from decreased or absent saliva production or decreased saliva density [8]. Dry mouth incidence rates following RAIT can range from 11% to 44% [9]. Dry mouth causes pain, difficulty in swallowing and speech, and taste changes [2, 8]. Dry mouth may also increase oral susceptibility sensitivity to infections such as dental caries and candidiasis [10]. Sialadenitis, dry mouth, or both may be developed immediately following RAIT or months later and may be transient or permanent [2, 11]. Persistency of these toxicities can negatively affect patients' quality of life [12].

Despite constant efforts to prevent and treat sialadenitis and dry mouth secondary to RAIT, prophylaxis and mitigation of these toxicities remain an unmet medical need. Drugs such as amifostine and pilocarpine have been used for this purpose but seem to be of limited efficacy [12]. An additional problem with these pharmacological interventions is that they sometimes induce severe side effects leading to poor adherence [12, 19].

The lack of comprehensive and effective management of sialadenitis and dry mouth induced by radioiodine treatment has led to the testing of other interventions to minimize salivary gland damage from radioiodine. According to the 2015 ATA thyroid nodule/differentiated thyroid carcinoma management guidelines [1], these “complementary and alternative procedures” include increased fluid intake and application of lemon candies, lemon juice, lemon slices, or chewing gum. However, according to these guidelines, there is no currently sufficient or consistent enough evidence to recommend in favor or against these interventions [1].

This judgment or rather lack of judgment prompted us to conduct the present systematic review of reports of clinical studies utilizing nonpharmacological interventions to reduce salivary gland damage following RAIT of DTC. Our aims included elucidating the current state of the art and identifying unmet research needs in this area.

## 2. Method and Materials

The study was undertaken between October 2014 and October 2015. A systematic search was performed in PubMed, CINHAL, Cochrane, and Scopus databases covering January 2000–October 2015. The keywords used were combinations of (AND, OR) “thyroid cancer”, “radioiodine therapy”, “salivary gland dysfunction”, “non-pharmacological methods”, and “lemon.”

Inclusion criteria were clinical studies that included adults (age > 18 years) who had received near-total thyroidectomy followed by RAIT. The studies had to use only nonpharmacological interventions to minimize salivary gland damage secondary to RAIT, and the reports had to be published in English. Bases for exclusion were discontinued studies, studies reported only in summaries, reviews, or meta-analyses, or studies including patients whose salivary gland damage was secondary to factors other than RAIT of DTC. Additionally, the bibliographies of the reviewed articles were checked to identify relevant clinical studies.

### 2.1. Methodological Assessment

All studies included in this study were reviewed individually by four authors (Andri Christou, Andreas Charalambous, Evridiki Papastavrou, and Anastasios Merkouris) using the Jadad scale of methodological quality [20]. The choice of Jadad was based on the scale's ease of use and inclusion of several components promoting trustworthiness and validity [21]. However, the scale score was not used as an inclusion criterion.

The Jadad scale grades 3 methodological qualities, with scores for each quality combined to produce an overall score of 0 to 5 points, with 0 being the lowest quality score and 5 being the highest. The parameters assessed include randomization (maximum of 2 points), blinding (maximum of 2 points), and participant exclusion (maximum of 1 point). Additionally, the TREND Statement Checklist, which is more detailed than the Jadad scale, was used to assess the included studies. The TREND Checklist consists of 22 sections divided into five categories, (1) title and abstract, (2) introduction, (3) methods, (4) results, and (5) discussion, to evaluate the quality of the papers regarding the trials [22].

## 3. Outcome of the Literature Search

The literature search yielded a total of 182 articles based on the predetermined keywords. Thirty-three articles were retrieved from PubMed, 140 from Scopus, 2 from CINHAL, and 7 from Cochrane.

**Figure 1:**
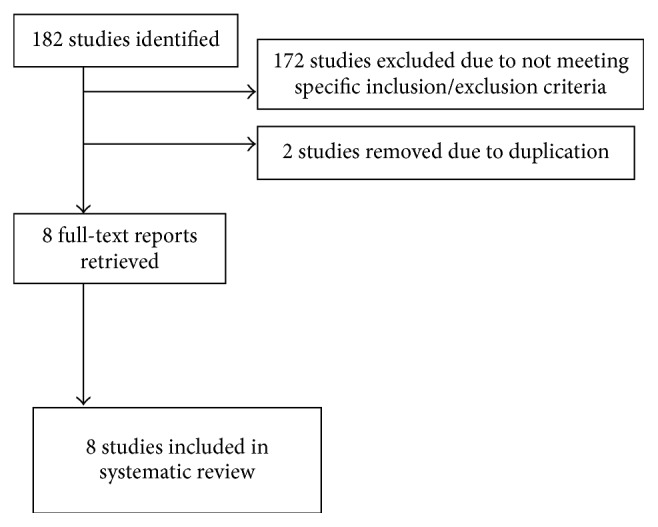
Literature search flow chart.

The researchers examined the identified articles to assess their conformity with the prespecified inclusion and exclusion criteria. One hundred seventy-two articles were excluded for not meeting the specific inclusion/exclusion criteria, and 2 additional papers were excluded for parallel suspension in two different databases. The remaining 8 articles were chosen to be further analyzed (Figure 1).

## 4. Results

Details regarding the included trials are displayed in Table 1.

The first report analyzed [9] was published in 2005 regarding a randomized controlled trial (RCT) in Japan, whereas the other 7 reports analyzed [13–18, 23] were published between 2010 and 2014, and the reported studies were conducted in China, Iran, United States, Republic of Korea, and Germany, respectively. The methodological assessment of the studies based on the Jadad scale showed that one study was given a grade of 1 [9], five studies were given a grade of 2 [15–18, 23], and two studies were given a grade of 3 [13, 14]. Problems with randomization, blinding, and high participant dropout were identified for all studies.

The trial [9] conducted in Japan (Hokkaido) was a nonrandomized trial with two dissimilar unequal groups, using measurements collected at an unspecified times frequency. Group A consisted of 105 participants who were given radioiodine from August 1999 to October 2000, whereas Group B consisted of 125 participants receiving the radioisotope from November 2000 to June 2002. The two groups differed regarding the timing, quantity, and frequency of lemon candy administration. Group A was given 1-2 lemon candies immediately after administration of 2.66–5.55 GBq of ^131^I and then every 2-3 hours, daily for 5 days. Group B received the same regimen as Group A did starting 24 hours later. The study variables, sialadenitis, taste dysfunction, and dry mouth, were assessed with a patient-completed questionnaire, about which no details were reported, a patient-completed visual analogue scale (VAS) as a subjective measure, and salivary gland scintigraphy as an objective measure. These assessments, whose numbers were not specified in the paper, were made during the treatment period, as well as every 1–6 months for a 24-month period. For the statistical analysis, *γ* ^2^ test and *t*-test were performed, using *p* < 0.05 to indicate statistically significant findings.

The results showed that sialadenitis, taste dysfunction, and dry mouth were much more intense in Group A than in Group B, that is, in patients receiving candy more frequently. Sialadenitis incidence was 63.8% in Group A, but 36.8% in Group B (*p* < 0.001). Taste dysfunction incidence was 39.0% in Group A, but 25.6% in Group B (*p* < 0.01). Dry mouth occurred in 23.8% of Group A, but in 11.2% of Group B (*p* < 0.005). Also, permanent dry mouth was reported in 14.3% of Group A, but in only 5.6% of Group B (*p* < 0.05). This study thus showed that lemon candy administration 24 hours following radioiodine ingestion provided better salivary gland-related outcomes compared to earlier and more frequent administration. The researchers hypothesized that administering lemon candy in the first 24 hours following radioiodine therapy resulted in more radioiodine absorption, exacerbating salivary gland damage, compared to giving candy later.

The next trial that we reviewed dealt with the effectiveness of vitamin C in decreasing ^131^I absorption by the salivary glands. This study was conducted by Liu et al. [13] in West China Hospital, in Chengdu, China. This study included 72 patients who underwent thyroidectomy and were given 3.7 GBq ^131^I from October 2006 to December 2007. The intervention included a lozenge containing 100 mg of vitamin C that was administered for sucking every 4 hours for 6 days, always following radioiodine consumption. The participants were separated into four groups according to the starting times of the vitamin C administration. Group A (18 participants) began sucking vitamin C 1 hour, Group B (18 participants) 5 hours, Group C (19 participants) 13 hours, and Group D (17 participants) 25 hours following RAIT. The assessment of salivary gland damage was done using salivary gland scintigraphy 1, 2, 3, 4, 5, 13, 25, and 48 hours after ^131^I administration. For the statistical analysis, *γ* ^2^ test and Kruskal-Wallis test were used, along with a *p* value of <0.05. The results showed no statistically significant difference in salivary damage among the study groups. Within-group analysis revealed that vitamin C had a limited effect in all groups, based on the scintigraphy measurements.

A prospective randomized study conducted by Kim et al. [16], in the Republic of Korea, aimed to assess the effect of parotid gland (PG) massage on PG Tc-99m pertechnetate uptake with *n* = 60 patients. Patients were randomly assigned into two groups (the first group, *n* = 30, received PG massage; the second group, *n* = 30, was a control group). Further, the groups were divided into two subgroups: the first group, *n* = 18 patients, classified as group Hyper-Mas (hyperthyroidism) and the second group, *n* = 12 patients, classified as group Euthy-Mas (normal thyroid function). In the control group, *n* = 19 patients were classified as group Hyper-noMas (hyperthyroidism) and *n* = 11 patients were classified as group Euthy-noMas (normal thyroid function).

A scan was performed 50 minutes following the administration of 185 MBq of Tc-99m pertechnetate. Following the scan, the patients in the first group received PG massage, 20 times over one minute, and then a second scan was performed. Regions of interest (ROIs) were marked on the scans to calculate total counts on PGs and accumulation ratios. For statistical analysis, *t*-test and Fisher exact test were used, along with *p* < 0.05. The study showed that there was no significant difference in the mean PG counts between the scans of the patients of the first group who received massage (8556.9 ± 3333.4 counts versus 8598.3 ± 3341.3 counts, *p* = 0.39), whereas the second image of the control group showed significantly higher mean PG count compared to the first image (8581.2 ± 3618.0 counts versus 9096.4 ± 3654.0 counts, *p* < 0.01). In addition, the mean accumulation ratio was found to be lower in the massage group compared to the control group (0.5% ± 3.3% versus 6.8% ± 3.8%, *p* < 0.001).

At this point, it is worth mentioning that, according to the results, younger patients who received massage were more likely to have negative accumulation (43.3% versus 0%, *p* < 0.01). On the other hand, younger patients in the control group were more likely to have positive accumulation (45.5 ± 12.9 years versus 51.1 ± 10.0 years, *p* = 0.09). Furthermore, it was observed that there was no significant statistical difference between the members of the four subgroups (hyperthyroid and normal thyroidism, 8598.3 ± 3341.3 counts versus 9096.4 ± 3654.0 counts, *p* = 0.584). The researchers concluded that massage can have a positive effect in patients who receive Tc-99m pertechnetate.

Fallahi et al. [14] in a double-blind RCT conducted from June 2006 to February 2007 tested the effectiveness of vitamin E in preventing salivary gland damage caused by ^131^I therapy in DTC patients following thyroidectomy. Performed at Tehran University in Iran, this study involved 36 participants who were divided into two groups: the experimental group (*n* = 19) and the control group (*n* = 17). The experimental group received 800 IU of vitamin E orally once a day for 1 week before radioiodine consumption, as well as for 4 weeks afterwards. Patients in the control group received placebo according to the same schedule. Participants in both groups underwent salivary gland scintigraphy on the day of administration of 3700–5500 MBq ^131^I and 6 months later. For the statistical analysis, Mann-Whitney *U* test, Fisher's exact test, and Wilcoxon Signed-Rank test were used, along with a *p* value of <0.10 for significance.

In the experimental group, there was no statistically significant difference before and after iodine consumption (*p* = 0.06–0.09), whereas in the control group there was greater damage of the right submandibular gland (maximum secretion percentage [*p* = 0.039] and excretion fraction [EF] [*p* = 0.015]) and the left parotid gland [EF] [*p* = 0.035] after RAIT. When the two groups were compared, only 7.9% of the experimental group compared to 26.5% of the control group had a >15% decrease in salivary gland EF (*p* = 0.035). Therefore, this study showed that vitamin E was effective in protecting the salivary glands.

A pilot study by Kulkarni et al. [15], which took place in Washington DC in the US, aimed to examine the radiation absorption by the parotid glands using time-activity curves (TACs). However, the main difference for Kulkarni et al.'s study was the fact that the patient did not receive a therapy dose but they just examined the uptake with a diagnostic dose.

Nine patients awaiting ^131^I ablation were included in the study. The patients underwent two salivary gland scintigraphies, the first just after drinking lemon juice and the second without lemon juice, after administration of 37–185 MBq of ^123^I. The two scintigraphies were performed within 8 days. A 37% decrease in radiation absorption was observed when patients took the lemon juice relative to when they did not take the juice. Based on the TACs, there was an increase in absorption beginning during the “no lemon juice” scan, an increase which lasted for 2 hours. The authors suggested that patients treated with ^131^I should be immediately and frequently given lemon juice to reduce salivary gland damage.

Hong et al. [17] conducted a prospective study in the Republic of Korea, aiming to examine the effect of massage on parotid gland radioiodine content with *n* = 44 patients who underwent total thyroidectomy followed by ^131^I therapy. The patients were divided into two groups: A and B.

Patients in both groups were given 18.5 Mbq ^123^I orally followed by a salivary scan two hours later. One minute after the first scan, patients in Group A were scanned again for control, received one-minute massage, and were scanned for the third time. Patients in Group B were scanned for control two minutes after the first scan, received two-minute massage, and were scanned for the third time. Regions of interest (ROIs) were marked on salivary scans and changes in the uptake of ^123^I at control (Δ*U*_con_) and changes in the uptake of ^123^I at massage (Δ*U*_mas_) were calculated. To statistically analyze the results, the researchers used the *F*-test and the *t*-test to compare Δ*U*_con_ and Δ*U*_mas_. The *p* value was set to *p* < 0.05.

The results indicated that the mean value of Δ*U*_mas_ was significantly lower than that of Δ*U*_con_ for patients in both groups (*p* < 0.001), but there was no significant difference between Δ*U*_mas_ (*p* = 0.573) and Δ*U*_con_ (*p* = 0.822) in the two groups. Therefore, researchers concluded that PG massage regardless of duration, one or two minutes, was helpful for patients who receive ^123^I as it can decrease radiation on the parotid glands following radioiodine therapy.

A prospective study conducted by Jentzen et al. [23] aimed to evaluate the effect of chewing lemon slices on absorbed doses to the salivary gland through the calculation of organ absorbed doses following the administration of radioiodine (ODpAs). The ODpAS in the nonstimulation group was compared against the ODpAs (stimulation group) in a previous study done by Jentzen et al. [24]. Both studies used six PET scans at 0.5, 1, 2, 4, 48, and ≥96 h and one PET/CT scan (ROIs) at 24 h after the administration of radioiodine, ^131^I in the first study and ^123^I in the second study by Jentzen et al. [23] (22.6–30.5 MBq), to *n* = 10 different patients. Blood samples were also collected at about 2, 4, 24, 48, and 96 h. However, in the first study [24], *n* = 10 patients were given lemon slices about 20 minutes after the administration of ^124^I and chewed on them over the first day (stimulation group). In the second study by Jentzen et al. [23], the patients were not given lemon slices (nonstimulation group). The researchers used the Kolmogorov-Smirnov test to check normality, the mean ± standard deviation, and Mann-Whitney *U* test. To compare the two studies Pearson's and Spearman's correlation coefficients were used. *p* for both studies was set to <0.05.

According to the comparison of the two studies, chewing on lemon slices shortly after the administration of ^131^I and ^124^I can increase radiation absorption. Explicitly, ODpA was lower in the nonstimulation group (0.23 Gy/GBq) than ODpA in the stimulation group (0.32 Gy/GBq). Moreover, it was found that the average blood uptake was similar in the stimulation and nonstimulation groups.

Similarly, in a prospective study, Jentzen et al. [18], in Germany, assessed the effectiveness of chewing gum stimulation on the absorbed doses to the salivary glands ^124^I PET/CT and the comparison with the results of a previous study [23], with *n* = 10 patients awaiting radioiodine therapy following total thyroidectomy. Those patients were given tasteless gum about 20 minutes after swallowing a 23 MBq ^124^I capsule. Also, three PET/CT scans 4, 24, and ≥96 h after the consumption of ^124^I were performed. Statistical analysis was performed using the mean, the median, and the SD and the Mann-Whitney *U* test as well as *p* < 0.05. The results of the current study were compared with the previous study [23], using Spearman's rank correlation coefficient.

The results showed that patients who chewed on lemon slices had higher blood flow increase than the patients who chewed gum (*p* < 0.04). Furthermore, patients who chewed gum had a little higher blood flow increase than the nonstimulation patients (*p* > 0.60). However, chewing tasteless gum immediately after administrating ^124^I did not significantly reduce the absorbed dose to the salivary glands; therefore, it cannot be considered effective (*p* = 0.99).

## 5. Discussion

This review sought to examine the use of nonpharmacological methods in the prevention and mitigation of salivary gland damage following RAIT in patients with DTC. At this point, it is essential to clarify the function of salivary glands, in order to support the mechanism which may cause their damage. Indeed, the toxic effect of ^131^I caused to the salivary glands is quite serious due to their ability to absorb high levels of iodine compared to other tissues [6].

The salivary glands' main function is saliva secretion which helps in lubricating the food, digestion, immunity system, and homeostasis of the body. This damage appears in the endothelial blood vessels of the gland. It increases vascular permeability and produces escape of proteins and electrolytes, so plasma proteins enter the saliva and sodium and chloride produced from acinar cells are normally transported in the saliva [7]. However, irradiated duct due to ^131^I cannot resorb in the saliva. Finally, biochemical changes can be noticed in the saliva (high sodium and chloride and also low phosphate) [7]. On the other hand, the positive effect of the sialogogues (e.g., lemon candy, lemon juice) and massage increase salivary secretion and as a consequence sialogogues help the excretion of the radioiodine.

The studies analyzed recommended that methods such as vitamin E, vitamin C, lemon candy, lemon juice, and parotid massage use can help minimize salivary gland damage. Collectively, the studies examined agreed that sialogogues are effective; however, there was no consensus on the administration protocol, specifically the starting time and frequency of the intervention. For example, Nakada et al. [9] suggested that lemon candy should be given 24 hours after radioiodine administration. This finding coincides with Jentzen et al.'s [23, 24] suggestion that the intake of lemon juice should be delayed and it should be taken after 24 hours because when it is immediately taken after radioiodine it increases the absorbed dose from the salivary glands. Contrary to Nakada et al. [9] and Jentzen et al. [18, 23], Kulkarni et al. [15] followed a different administration protocol, by which lemon juice was administered immediately following ^123^I ingestion. According to Liu et al. [13], sialogogue administration time was not important, since vitamin C was reported to have effects in all groups. Fallahi et al. [14], on the other hand, focused on the effect of vitamin E without examining administration time or frequency, perhaps suggesting that the administration time would not affect the results.

As far as the effect of massage on the parotid glands is concerned, this was examined in two studies [16, 17] and in two different types of radioiodine (^123^I versus Tc-99m pertechnetate). The two studies showed that massage has an effect on the salivary accumulation and excretion of radioiodine. Explicitly, Hong et al.'s [17] study concluded that PG massage regardless of duration, one or two minutes, was helpful for patients who receive ^123^I as it can decrease radiation accumulation on the parotid glands following radioiodine therapy. Similarly, Kim et al. [16] concluded that PG massage has a positive effect on patients that receive Tc-99m pertechnetate, by reducing the absorbed doses by the glands following administration of ^131^I for DTC.

The reviewed studies provided encouraging results for the effectiveness of nonpharmacological interventions; however, these should be read in light of some limitations. To start with, four of the studies [9, 13, 15, 23] assumed that sialogogues can prevent radioiodine-induced salivary gland damage, and their aim was to examine the best timing of administration of the agents, that is, lemon candy, vitamin C, lemon juice, or lemon slice [23], and not to examine whether these agents were actually effective. Two studies [16, 17] were linking parotid gland massage with positive effects on the decrease of the absorption of radioiodine. However, another study [18] supported the notion that taking chewing gum directly after radioiodine had no significant reduction on the absorption from the salivary glands.

Also, there have been trials on the effect of sialogogues on patients with dry mouth resulting from other treatments (e.g., chemotherapy) and causes such as Sjögren's syndrome [12]. However, these trials furnished no scientific evidence that sialogogues alone are helpful in preventing sialadenitis and dry mouth in DTC patients receiving radioiodine therapy [12].

The literature on salivary gland damage secondary to ^131^I treatment of DTC also highlights the notion that efforts to minimize such damage have included use of pharmacological interventions such as amifostine or pilocarpine. A study by Haddad et al. [25] showed that patients with locally advance head and neck cancer who were given chemotherapy or radiotherapy did not have better dry mouth grade or saliva secretion or less salivary gland swelling with than without amifostine. Application of pilocarpine as a protective factor for the management of dry mouth has shown mixed results regarding efficacy [12]. A systematic review conducted by Jense et al. [26] supports the notion that chemotherapy causes severe damage to the salivary glands, whereas nonpharmacological methods may have positive effect on salivary glands; thus, iodine is causing less damage. Therefore, increasing or stimulating salivation by administration of sialogogues will be more effective in reducing ^131^I transit in the gland and reducing the damage.

In addition, a comparative study showed a statistically significant difference regarding saliva secretion from the parotid glands in patients taking pilocarpine versus placebo [27]. In a different study, though, saliva production in those taking pilocarpine was less than that seen in patients receiving the submandibular gland transfer method which is a surgical intervention [28].

Thus, researchers examining the use of pharmacological methods such as pilocarpine and amifostine for protecting the salivary glands of DTC patients given ^131^I also coadministered sialogogues according to ATA instructions. Aframian et al. [29] state that pilocarpine increased salivary excretion and Bohuslavizki et al. [30] affirm that amifostine decreased salivary gland damage, hence improving patient quality of life. Nevertheless, these investigators attributed the positive results of the trials only to pilocarpine or amifostine, and they did not examine whether or to what extent sialogogues contributed to the results. Thus, safe conclusions about the effectiveness of sialogogues cannot be made based on these combination studies.

The studies reviewed in the present paper need to be interpreted in light of specific limitations. Specifically, problems were identified regarding sample size, randomization methods, and assessment frequency. Only Nakada et al.'s study [9] had a large number of participants (*n* = 230), while the others had sample sizes ranging from 9 to 72 [13–18, 23]. In Nakada et al.'s study [9], patients were allocated to treatment groups based on their date of presentation, that is, groups comprising consecutive patients treated at different periods of different lengths. Additionally, the paper does not provide further details on treatment assignment. There was no randomization, reducing the intrarater reliability and increasing the likelihood of selection bias. In addition, the paper never references the blinding of subjects, increasing the chance of statistical error. In the studies by Liu et al. [13] and Fallahi et al. [14], on the other hand, although there was randomization and there was blinding, how the blinding was accomplished was not explained in the respective publications. Finally, Kulkarni et al. [15] report that randomization, but not blinding of participants, was performed. It is worth noting that all eight trials were single-site, therefore limiting the generalizability of their results.

Another difficulty in comparing the results of the analyzed studies lies in the different radioiodine activities and isotopes used. However, one study [15] was on patients that did not receive therapy and, therefore, this issue might affect the results in this literature review. The literature supports the notion that these factors can affect the degree of sialadenitis and dry mouth; thus, these characteristics of the reviewed studies should be considered in interpreting their results [31]. Additionally, none of the reviewed papers described which healthcare professionals provided instructions to patients on use of the studied sialogogues or how such instructions were provided or in what form (e.g., verbally in person or by telephone or in writing).

All eight studies used a variety of objective measures that increased the validity of their findings. Liu et al. [13], Nakada et al. [9], and Fallahi et al. [14] used salivary gland scintigraphy using intravenous Tc-99m Tc pertechnetate, and Kulkarni et al. [15] used salivary gland scintigraphy using ^123^I. In addition to scintigraphy, Nakada et al. [9] used a questionnaire and a VAS scale, as subjective measurements, without providing details on these instruments or how they were administered.

Finally, the analyzed studies provided no information on ethical issues or participant recruitment flow charts. Consequently, our review should take into account these limitations, which could threaten the reliability and the validity of the included studies' findings.

This review could have been broader, covering types of research other than clinical trials as well as including papers published in languages other than English. Furthermore, this review could not be a meta-analysis, due to the fact that the number of articles on this topic identified as suitable for analysis was small, and these papers received only average scores on the Jadad scale. It is hoped and expected that, in the future, more clinical trials will take place, resulting in a suitable amount of evidence for meta-analysis.

## 6. Conclusion

Based on the available evidence, sialogogues such as lemon candy, vitamin E, lemon juice, and lemon slice and parotic gland massage can reduce the damage on salivary glands. Lemon candy appears to have the most statistically positive significant effect from all the nonpharmacological interventions studies in this review. The small number of clinical studies about the effectiveness of nonpharmacological methods in the reduction of salivary gland damage in DTC patients receiving RAIT indicates that such interventions have not been sufficiently or systematically investigated. This observation supports the classification of this evidence by the ATA guidelines committee. However, the fact that one of the trials took place in 2005-2006 and seven between 2010 and 2014 suggests that interest in this area may be growing. Despite the encouraging nature of the studies' findings, the limitations regarding the small sample sizes and the documentation of results point towards the need for further, more systematic clinical research about the use of nonpharmacological methods to prevent or decrease salivary gland damage and thereby improve the quality of life of DTC patients after RAIT. It also should be emphasized that the results of trials of pharmacological interventions are controversial, since researchers disagree about the effectiveness of the studied drugs and the published studies have shown that these agents have undesirable side effects. Since the combination of nonpharmacological and pharmacological interventions together is being practiced, clinical studies about the effect of nonpharmacological methods alone are essential.

## Figures and Tables

**Table 1:** 

Authors, country, year of publication	Study design	Assessment methods and times	Radioiodine activity	Statistical analysis	Results	Jadad^1^ score
Nakada et al., Japan, 2005 [9]	105 participants receiving ^131^I therapy in control group were given 1-2 lemon candies immediately after iodine consumption and then every 2-3 hours for 5 days 125 participants in experimental group had the same intervention, but starting 24 hours after iodine ingestion Allocation was not randomized; each study group's members were recruited consecutively during different periods	Questionnaire and VAS Salivary gland scintigraphy with 99^m^ Tc-pertechnetate during hospitalization and after 1–6 months for 24-month duration	3.66 GBq versus 5.55 GBq between two groups	Chi-squared test, *t*-test (*p* < 0.05)	Sialadenitis incidence in Group A = 63.8% and in Group B = 36.8% (*p* < 0.001) Taste dysfunction incidence in Group A = 39.0% and in Group B = 25.6% (*p* < 0.01) Dry mouth incidence in Group A = 23.8% and in Group B = 11.2% (*p* < 0.005) Dry mouth in Group A = 14.3% and 5.6% in Group B (*p* < 0.05)	1

Liu et al., China, 2010 [13]	72 participants receiving ^131^I therapy Patients sucked vitamin C lozenge (100 g) every 4 hours for 6 days starting either 1 hour (*n* = 18), 5 hours (*n* = 18), 13 hours (*n* = 19), or 25 hours (*n* = 17) after ^131^I consumption	Salivary gland scintigraphy with 99^m^ Tc-pertechnetate 1, 2, 3, 4, 5, 13, 25, and 48 hours after ^131^I	3.7 GBq	Chi-squared and Kruskal-Wallis test (*p* < 0.05)	Salivary gland cumulative activities from the first 24 hours after ^131^I administration accounted for 86.08% ± 7.89% No statistically significant differences among the 4 groups Vitamin C had limited effect in all groups *p* = 0.37	3

Fallahi et al., Iran, 2013 [14]	36 participants receiving ^131^I therapy 19-patient experimental group was given 800 IU vitamin E/day for 1 week before radioiodine administration, as well as the 4 weeks afterwards 17-patient control group was given a placebo according to the same regimen	Salivary gland scintigraphy with 99^m^ Tc-pertechnetate was performed on the day of RAIT as well as 6 months later (follow-up) Salivary gland excretion function was measured	3700–5550 MBq	Mann-Whitney *U* test Fisher's Exact test Wilson Signed-Rank test *p* < 0.10	Experimental group: no statistical difference before versus after iodine Control group: decline in the amount of the parotid gland uptake after iodine In the experimental group, there was a change in the first minute uptake ratio in the right parotid gland and the excretion function of the left parotid gland suffered less damage Salivary gland excretion experimental group = 7.9% versus 26.5% for control group, with more than 15% absolute decrease in the experimental group (*p* = 0.035)	3

Kulkarni et al., USA, 2014 [15]	9 participants awaiting ^131^I therapy: prospective pilot study	2 salivary gland scans performed over 8 days One salivary gland scintigraphy done with lemon juice (SSwLJ) after ^123^I administration Second salivary scintigraphy done without lemon juice (SSwoLJ), again after ^123^I administration Examine the potential absorbed dose in parotid glands with the use of TAC	37 MBq–185 MBq (^123^I) (dose of iodine given only for the purpose of scintigraphy and not for therapy)	Descriptive statistics Wilcoxon Signed-Rank sum test *p* < 0.0001	37% decrease in radiation absorbed dose in salivary glands Mean TAC = 70749.4 (SD = 53516.5, median = 51258) for SSwoLJ Mean TAC = 43638.6 (SD = 310227.7, median = 29887) for SSwLJ Area under TAC = 27110.8 (SD = 25808.8, median = 16669) for SSwoLJ Significantly higher for SswoLJ	2

Kim et al., Republic of Korea, 2012 [16]	60 participants initially divided into 2 equal groups The patients in the 1st group received parotid gland (PG) massage 18 of them were classified as group Hyper-Mas (hyperthyroidism) and 12 as group Euthy-Mas (normal thyroid function) PG massage 20 times over 1 min The patients in the 2nd group did not receive PG massage 19 were classified as group Hyper-Mas (hyperthyroidism) and 11 as group Euthy-No Mas (normal thyroid function)	Salivary scans (ROIs) Total counts of both PGS and accumulation were calculated	185 MBq of Tc-99m pertechnetate	Mean ± SD, *t*-test, and Fisher exact test *p* < 0.05	PG counts and accumulation ratios PG count on first image Group Hyper-Mas = 7563.7 ± 2962.4 Group Hyper-noMas = 8618.7 ± 3173.8 *p* = 0.891 PG count on second image Group Hyper-Mas = 7615.0 ± 3010.0 Group Hyper-noMas = 9188.0 ± 3152.8 *p* = 0.781 Accumulation ratio (%) Group Hyper-Mas = 0.7 ± 3.2 Group Hyper-noMas = 7.4 ± 3.8 *p* < 0.01 No statistical difference between group Euthy-Mas and group Euthy-noMas *p* = 0.879 *p* = 0.801 *p* = 0.002	2

Hong et al., Republic of Korea, 2014 [17]	44 participants Total thyroidectomy followed by ^131^I therapy Group A and Group B A salivary scan two hours after ^123^I administration. One minute later, patients in Group A were scanned again for control, received one-minute massage, and were scanned for the third time Patients in Group B were scanned for control two minutes after the first scan, received two-minute massage, and were scanned for the third time	Three salivary scans, ROIs, and Δ*U*_con_ and Δ*U*_mas_	18.5 Mbq ^123^I orally	*F*-test, *t*-test to compare Δ*U*_con_ and Δ*U*_mas_ *p* < 0.05	The mean value of Δ*U*_mas_ is significantly lower than Δ*U*_con_ for patients in both groups (*p* < 0.001) No significant difference between Δ*U*_mas_ (*p* = 0.573) and Δ*U*_con_ (*p* = 0.822) in the two groups	2

Jentzen et al., Germany, 2010 [23]	Two studies, with 10 participants in each study In the first study, ten patients were given lemon slices about 20 minutes after administration of ^124^I; they chewed on them over the first day (stimulation group) In the second study, patients were not given lemon slices (nonstimulation group)	Both studies used six PET scans at 0.5, 1, 2, 4, 48, and ≥96 h and one PET/CT scan (ROIs) at 24 h after the administration of radioiodine Blood samples were collected at about 2, 4, 24, 48, and 96 h	^131^I in the first study and ^124^I in the second study (22.6–30.5 MBq)	Kolmogorov-Smirnov test Mean ± standard deviation Mann-Whitney *U* test Pearson's and Spearman's correlation coefficients were used *p* < 0.05	ODpA was 0.23 Gy/GBq in the nonstimulation group and 0.32 Gy/GBq in the stimulation group Average blood uptake similar in the stimulation and nonstimulation groups	2

Jentzen et al., Germany, 2014 [18]	*n* = 10 patients awaiting radioiodine therapy after total thyroidectomy. Those patients were given tasteless gum about 20 minutes after swallowing a 23 MBq ^124^I capsule	Three PET/CT scans 4, 24, and ≥96 h after the consumption of ^124^I were performed	23 MBq ^124^I capsule	Statistical analysis was performed using the mean, the median, and the SD and the Mann-Whitney *U* test as well as *p* < 0.05	The current study showed that patients who chewed on lemon slices had a higher blood flow increase than the patients who chewed gum (*p* < 0.04). Patients who chewed gum had a little higher blood flow increase than the nonstimulation patients (*p* > 0.60). However, chewing tasteless gum immediately after administering ^124^I did not significantly reduce the absorbed dose to the salivary glands; therefore, it cannot be considered effective (*p* = 0.99)	2

SSwoLJ: salivary gland scintigraphy without lemon juice; SSwLJ: salivary gland scintigraphy with lemon juice; TAC: time-activity curve; Δ*U*_con_: changes in the uptake of ^123^I at control; Δ*U*_mas_: changes in the uptake of ^123^I at massage; ODpAs: organ absorbed doses.

^1^The Jadad scale grades 3 methodological qualities, with scores for each quality combined to produce an overall score of 0 to 5 points, with 0 being the lowest quality score and 5 being the highest. The parameters assessed include randomization (maximum of 2 points), blinding (maximum of 2 points), and participant exclusion (maximum of 1 point).
